# FAM83H‐AS1 is a noncoding oncogenic driver and therapeutic target of lung adenocarcinoma

**DOI:** 10.1002/ctm2.316

**Published:** 2021-02-14

**Authors:** Siwei Wang, Chencheng Han, Tongyan Liu, Zhifei Ma, Mantang Qiu, Jie Wang, Qingjun You, Xiufen Zheng, Weizhang Xu, Wenjia Xia, Youtao Xu, Jingwen Hu, Lin Xu, Rong Yin

**Affiliations:** ^1^ Department of Thoracic Surgery, Jiangsu Key Laboratory of Molecular and Translational Cancer Research Nanjing Medical University Affiliated Cancer Hospital & Jiangsu Cancer Hospital & Jiangsu Institute of Cancer Research Nanjing China; ^2^ Department of Science and technology Nanjing Medical University Affiliated Cancer Hospital & Jiangsu Cancer Hospital & Jiangsu Institute of Cancer Research Nanjing China; ^3^ Department of Thoracic Surgery Peking University People's Hospital Beijing China; ^4^ Biobank of Lung Cancer Jiangsu Biobank of Clinical Resources Nanjing China; ^5^ Department of Thoracic Surgery The Affiliated Hospital of Jiangnan University Wuxi China; ^6^ Collaborative Innovation Center for Cancer Personalized Medicine Nanjing Medical University Nanjing China

**Keywords:** driver gene, long noncoding RNA, lung adenocarcinoma, therapeutic target

## Abstract

**Background:**

Little is known about noncoding oncogenes of lung adenocarcinoma (LUAD), and these potential drivers might provide novel therapeutic targets.

**Methods:**

Since abnormally overexpression of oncogenic drivers is induced by genomic variation, we here utilized genomic, transcriptomic, and clinical prognosis data of The Cancer Genome Atlas (TCGA) LUAD datasets to discover novel drivers from long noncoding RNAs. We further used zebrafish models to validate the biological function of candidates in vivo. The full length of FAM83H‐AS1 was obtained by rapid amplification of the cDNA ends assay. RNA pull‐down, RNA immunoprecipitation, quantitative mass spectrometry, and RNA sequencing assays were conducted to explore the potential mechanisms. Additionally, we used CRISPR interference (CRISPRi) method and patient‐derived tumor xenograft (PDTX) model to evaluate the therapeutic potential of targeting FAM83H‐AS1.

**Results:**

The results suggest that FAM83H‐AS1 is a potential oncogenic driver due to chromosome 8q24 amplification. Upregulation of FAM83H‐AS1 results in poor prognosis of LUAD patients in both Jiangsu Cancer Hospital (JSCH) and TCGA cohorts. Functional assays revealed that FAM83H‐AS1 promotes malignant progression and inhibits apoptosis. Mechanistically, FAM83H‐AS1 binds HNRNPK to enhance the translation of antiapoptotic oncogenes RAB8B and RAB14. Experiments using CRISPRi‐mediated xenografts and PDTX models indicated that targeting FAM83H‐AS1 inhibited LUAD progression in vivo.

**Conclusions:**

Our work demonstrates that FAM83H‐AS1 is a noncoding oncogenic driver that inhibits LUAD apoptosis via the FAM83H‐AS1–HNRNPK–RAB8B/RAB14 axis, which highlights the importance and potential roles that FAM83H‐AS1 may serve as a novel therapeutic target for LUAD.

## Graphical abstract

Integrated bioinformatic analysis and in vivo and in vitro experiments revealed that long noncoding RNA FAM83H‐AS1 is a novel driver in lung adenocarcinoma (LUAD). FAM83H‐AS1 inhibits LUAD apoptosis via the FAM83H‐AS1–HNRNPK–RAB8B/RAB14 axis and could serve as a novel therapeutic target for LUAD.

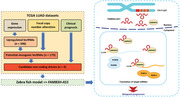

## 1 INTRODUCTION

Identifying cancer driver genes is essential for precision oncology. Somatic mutations in driver genes have been revealed across multiple types of cancers,^1^, ^2^ and a number of these driver genetic alterations have become therapeutic targets or prognostic markers. Recently, somatic copy number alterations (SCNAs) have been found to affect a larger fraction of cancer genomes than any other type of somatic genetic alterations, and these frequently altered genomic regions have critical roles in activating and inactivating oncogenic pathways.^3^ In lung cancer, the TracerX program demonstrated that a high frequency of SCNAs, but not somatic mutations, is significantly correlated with a poor survival rate.^4^ Therefore, among the considerable number of genes located in SCNA regions, novel cancer drivers should be further investigated.

Long noncoding RNAs (lncRNAs) play critical roles in cancer development, and the expression levels of lncRNAs are closely associated with oncogenic functions. Several oncogenic lncRNAs, such as FAL1 and PRAL, have been found to be regulated by SCNAs.^5^, ^6^ According to expression profiles of matched clinical and SCNA data, oncogenic drivers of lncRNAs can be distinguished from passengers by mathematical methods.^7^ However, few lncRNAs have been identified as oncogenic drivers in lung adenocarcinoma (LUAD), which is the leading cause of cancer‐associated deaths worldwide and accounts for nearly 40% of all lung cancer cases.^8^ Therefore, systematic exploration and identification of noncoding drivers in LUAD is warranted. Since the expression levels of oncogenic lncRNAs have been thought to be regulated by corresponding genomic alterations,^7^ multidimensional data, including clinical prognosis, gene expression, and SCNAs, were used to detect novel drivers from oncogenic lncRNAs.

In the current study, we extracted SCNA, gene expression, and clinical prognosis data from The Cancer Genome Atlas (TCGA) LUAD datasets to identify novel drivers from oncogenic lncRNAs using multi‐omics methods. We characterized a highly expressed lncRNA FAM83H‐AS1, which was regulated by frequent 8q24 amplification and was associated with poor prognosis. The characteristics of FAM83H‐AS1 were subsequently validated in the independent cohort and additional public datasets. Experimental investigation revealed that FAM83H‐AS1 could bind heterogeneous nuclear ribonucleoprotein K (HNRNPK) and increase the protein levels of RAB8B and RAB14, thus suppressing apoptosis and promoting tumorigenesis of LUAD cells. Importantly, targeting FAM83H‐AS1 remarkably reduced LUAD growth in the patient‐derived tumor xenograft (PDTX) model.

## 2 MATERIALS AND METHODS

### 2.1 Identification of differentially expressed lncRNAs in LUAD

RNA sequencing (RNA‐Seq) data of the TCGA LUAD datasets were downloaded from the data portal (https://portal.gdc.cancer.gov) for 585 LUAD patients, including 56 normal lung tissue samples. The R package DESeq was applied to counts data,^9^ and it detected 7320 differentially expressed genes (fold change > 2 and false discovery rate [FDR] < 0.05) among 60,483 genes. According to the “Gene_type” annotation by the Ensembl genes database, 596 lncRNAs were screened from differentially expressed genes. To validate the results, two public datasets, GSE74095 and GSE12236, were obtained from Gene Expression Omnibus (GEO; https://www.ncbi.nlm.nih.gov/gds) for use.

### 2.2 SCNAs and clinical data of LUAD cases

The SCNA profiles of 513 LUAD patients of TCGA were obtained from the data portal (https://portal.gdc.cancer.gov). To estimate copy number alteration, we analyzed corresponding Affymetrix SNP 6.0 array data to identify repeated genomic regions and identified focal SCNA profiles using GISTIC algorithm.^10^ Clinical characteristics of TCGA LUAD patients, including overall survival (OS) and disease‐free survival, were obtained from cBioPortal (http://www.cbioportal.org). The independent GSE29065 and GSE28572 datasets and the related prognosis data were obtained from GEO, and the probe annotations for GSE29065 and GSE28572 were queried from GEO platforms.

### 2.3 LUAD tissue samples and microarrays

All primary LUAD tissues and adjacent normal tissues were collected from patients who had undergone surgery at the Department of Thoracic Surgery, Nanjing Medical University Affiliated Cancer Hospital (Jiangsu Cancer Hospital [JSCH], Nanjing, China). All included tissue samples were confirmed by experienced pathologists and conducted in accordance with the International Ethical Guidelines for Biomedical Research Involving Human Subjects. Written informed consent was obtained from all patients. This study was approved by the Ethics Committee of JSCH. Tissue microarray (TMA) was constructed as described previously.^11^ Sixty‐eight pairs of lung cancer tissues and adjacent normal tissues from JSCH cohort were used to construct the TMA. RNA chromogenic in situ hybridization (CISH) was performed to detect FAM83H‐AS1 expression in TMA using digoxigenin‐labeled probe (C10910 lnc1100151, RiboBio). According to percentages of positive stained cancer cells and areas, the CISH score was rated on a scale of 1–12 as described previously.^11^ The characteristic and prognostic information of patients included in this study was obtained from follow‐up team of JSCH.

### 2.4 Cell culture, cell proliferation, colony formation, and apoptosis assays

All cell lines (A549, H1299, H1650, SPC‐A1, H1975, H358, PC9, and human bronchial epithelial cell [HBE]) were purchased from Shanghai Institutes for Biological Science (Shanghai, China). A549, H1650, SPC‐A1, H1975, H358, and HBE were cultured in DMEM medium (KeyGene, Nanjing, China); H1299 and PC9 were cultured in RPMI1640 medium (KeyGene), supplemented with 10% FBS with 100 μg/mL penicillin and 100 mg/mL streptomycin included. All cell lines were grown in humidified air at 37°C with 5% CO_2_. Cell cultures were occasionally tested for mycoplasma (last tested December 2018). Authentication of cells was verified by short tandem repeat DNA profiling within 6 months, and cells used in experiments were within 10 passages from thawing. Cell proliferation was examined using a CCK‐8 Kit (Roche Applied Science). Colony formation assays were performed to monitor LUAD cell cloning capability. Flow cytometer (FACScan, BD Biosciences) equipped with CellQuest software (BD Biosciences) was used to detect apoptosis level.

### 2.5 RNA extraction, genome DNA extraction, nuclear and cytoplasmic fractions extraction, and real‐time quantitative PCR (qRT‐PCR) and western blot analysis

RNA extraction, DNA extraction, and qRT‐PCR were performed as described previously.^11^ GAPDH, β‐actin, and snRNA U6 were used as internal controls. All primer sequences were listed in Table S1. RNA and protein isolation of nuclear and cytoplasmic fractions were applied with using PARIS Kit according to the manufacturer's protocol (Ambion, Life Technologies). Protein was extracted from transfected cells and quantified as previously described.^12^ All antibodies were listed in Table S2.

### 2.6 Small interfering RNAs and plasmid construction and cell transfection

The small interfering RNAs (siRNAs) were provided by Realgene Biotechnology (Nanjing, China). The full‐length cDNA of human FAM83H‐AS1 was synthesized and cloned into the expression vector pCDNA3.1 by Vigene Bioscience (Jinan, China). The final construct was verified by Sanger sequencing. SiRNA and plasmid vectors transfection was performed as described previously.^11^ All siRNA sequences used are listed in Table S3.

### 2.7 Rapid amplification of cDNA ends

5′‐RACE (rapid amplification of cDNA ends), 3′‐RACE, and full‐length amplification of FAM83H‐AS1 were performed using a SMART RACE cDNA Amplification Kit (Clontech) according to the manufacturer's instructions. The gene‐specific primers used for RACE analysis are presented in Table S1.

### 2.8 RNA immunoprecipitation and pull‐down assays

RNA immunoprecipitation was performed as described previously,^11^ and magnetic beads were conjugated with anti‐HNRNPK (ABCAM) or control anti‐IgG (Millipore) antibody. In vitro translation assays were performed using mMESSAGE mMACHINE T7 Transcription Kit (Invitrogen) according to the manufacturer's instructions. Then, FAM83H‐AS1 RNAs were labeled with desthiobiotinylation using the Pierce RNA 3′End Desthiobiotinylation Kit (Thermo Fisher). RNA pull‐down assays were performed with Magnetic RNA‐Protein Pull‐Down Kit (Thermo Fisher) according to the manufacturer's instructions. After elution of RNA‐interacting proteins, they were subjected to mass spectrometric analysis. Liquid chromatography–mass spectrometry experiments were performed with a linear ion trap quadrupole mass spectrometer (Thermo Finnigan) equipped with a micro‐spray source.

### 2.9 Luciferase reporter assays

The mRNA internal ribosome entry segment (IRES) of RAB8B and RAB14 was predicted by IRESite (http://iresite.org). The HNRNPK‐binding sites of RAB8B and RAB14 mRNA were identified by the Blast program. The sequences of different fragments were synthesized and then inserted into the pGL3‐basic vector (Vigene Bioscience). All constructs were verified by Sanger sequencing, and luciferase activity was assessed using the Dual Luciferase Assay Kit (Promega) according to the manufacturer's instructions.

### 2.10 RNA‐Seq and quantitative mass spectrometry

A549 cells were plated in a six‐well plate and transfected with an siRNA targeting FAM83H‐AS1 or a negative control. Twenty‐four hours after transfection, cells were harvested for RNA extraction and subsequent library construction and sequencing (CapitalBio Technology, Beijing, China). We used Mapsplice to calculate the number of mapped reads to each gene and then utilized DESeq to analysis the differentially expressed genes (fold change > 2 and FDR < 0.05).^13^ Similarly, cells were harvested for protein extraction and subsequent iTRAQ (Isobaric Tag for Relative Absolute Quantitation)/TMT (Tandem Mass Tags) detection (PTM Bio, Hangzhou, China). The identification of differentially expressed proteins was conducted as described in the previous study,^14^ which considered the number of annotated peptide sequences. We performed enrichment analysis on RNA‐Seq and quantitative mass spectrometry (QMS) results using R package clusterProfiler.^15^

### 2.11 CRISPR interference‐mediated generation of FAM83H‐AS1 knockdown LUAD cells

For the CRISPR interference (CRISPRi) experiments, six paired small guide RNAs (sgRNAs) were designed to target the transcription start site (TSS) of FAM83H‐AS1 locus (Hg 19) within 250 bp upstream and downstream. The sgRNA oligos were designed, phosphorylated, annealed, and cloned into a pBHCas‐ZXS 023 vector using a BsmBI ligation strategy. Additional details and a list of the sgRNA sequences can be found in Table S1.

### 2.12 In vivo tumor growth assays, tumor engraftment, and PDTX maintenance

All animal experiments were approved by the Nanjing Medical Experimental Animal Care Commission. The zebrafish tumor model was constructed according to the previous study.^16^ In brief, anesthetized embryos were subjected for microinjection. Approximately 400 tumor cells of control or silenced group were labeled by CellTracker CM‐DiI (Invitrogen) and 5 nL of the cell solution was injected into the perivitelline space of each embryo by an microinjector. Non‐filamentous borosilicate glass capillaries needles were used for injection and the injected zebrafish embryos were immediately transferred into aquarium water. Zebrafish embryos were monitored at 96 h for investigating tumor proliferation and metastasis using a fluorescent microscope. BALB/c nude mice (4–6 weeks), purchased from the Vital River Laboratory Animal Technology (Beijing, China), were maintained under specific pathogen‐free conditions. For the tumor formation assay, 10^6^ CRISPRi‐constructed or control cells were subcutaneously injected into one flank of each mouse. Tumor volume was calculated using the following equation: *V* = 0.5 × *D* × *d* ^2^ (*V*, volume; *D*, longitudinal diameter; *d*, transverse diameter). The method of building PDTX model has been described in the previous study.^11^

### 2.13 Statistical analysis

R software version 3.5.1, GraphPad prism 8, and SPSS 23 were used to analyze data and plot the figures. Differences between groups were assessed by two‐tailed Student's *t* test. The strength of the association between continuous variables was tested using Pearson's correlation test. Uni‐ and multivariate Cox regressions were used to identify independent risk factors. For survival analysis, OS was calculated using the Kaplan–Meier method and the log‐rank test. All *p*‐values were two sided and *p*‐values < 0.05 were considered to be statistically significant.

## 3 RESULTS

### 3.1 Identification of novel drivers from oncogenic lncRNAs in LUAD

**FIGURE 1:**
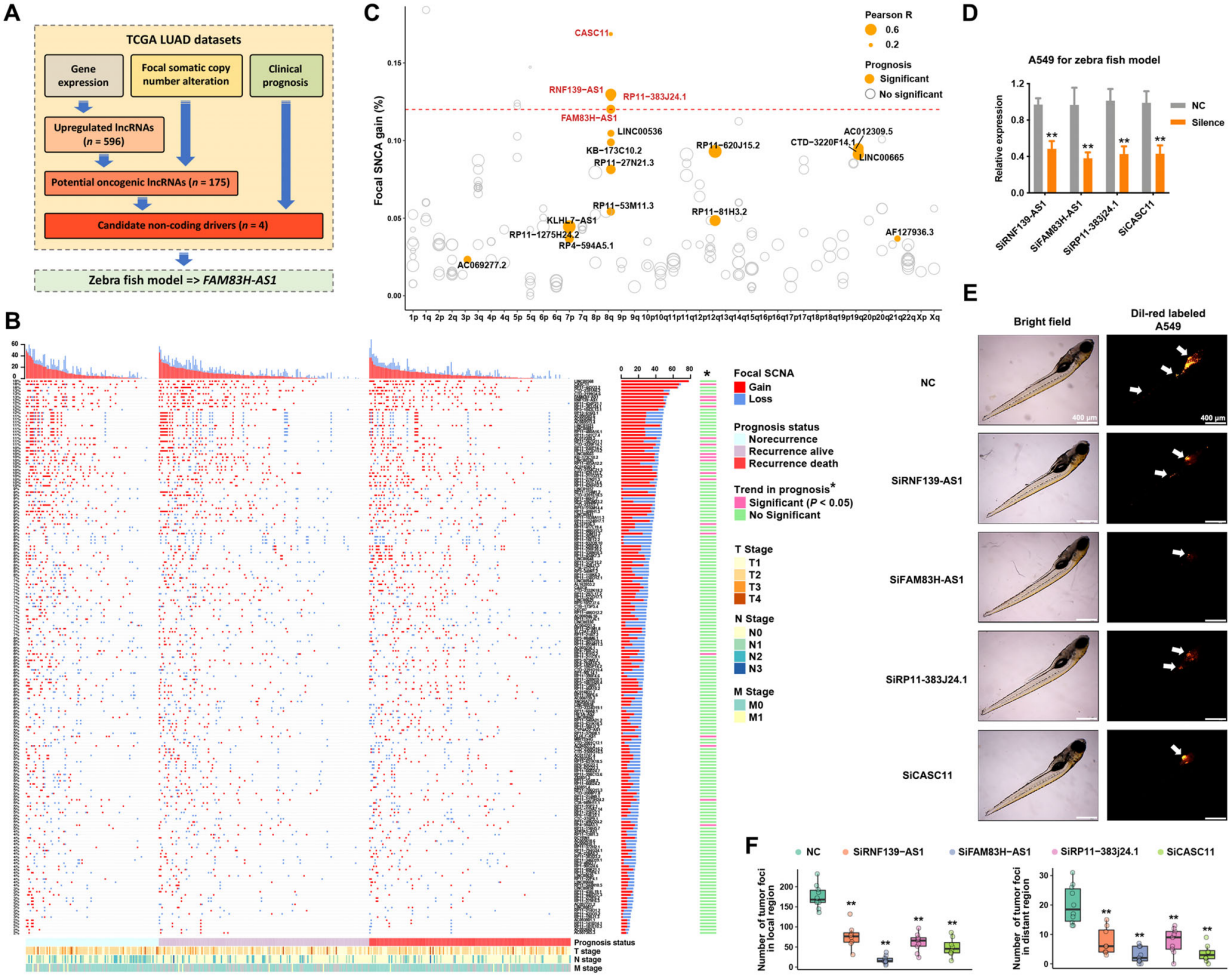
Identification of novel noncoding drivers from oncogenic lncRNAs in LUAD. (A) Screening of noncoding drivers revealed FAM83H‐AS1 as a potential driver gene of LUAD. Upregulated lncRNAs were identified according to TCGA LUAD RNA‐Seq data using DESeq method, and the analysis of focal SCNA was performed on TCGA LUAD genome‐wide segments data utilizing GISTIC algorithm. (B) Focal SCNA gain or loss: the GISTIC value is greater than 0.3 or less than –0.3. Heatmaps were separated into three parts according to different prognosis statuses, and the Chi‐squared test for trend was conducted. (C) Focal SCNA frequency, clinical relevance, and correlation between expression and focal SCNA levels of 175 potential oncogenic lncRNAs. (D) Silence efficiency of siRNAs in A549 cells, which were used for zebrafish assays. The siRNA of FAM83H‐AS1 was using siRNA2. (E and F) For the high‐ranking lncRNAs, DiI‐red‐labeled A549 cells in zebrafish embryos identified oncogenic functions that promoted proliferation and metastasis of RNF139‐AS1, FAM83H‐AS1, RP11‐383j24.1, and RP11‐429J17.7 in vivo (NC, *n* = 10 zebrafish/group; siRNF139‐AS1, *n* = 8 zebrafish/group; siFAM83H‐AS1, *n *= 9 zebrafish/group; siRP11‐383j24.1, *n* = 9 zebrafish/group; siCASC11, *n* = 8 zebrafish/group). *p < 0.05 and **p < 0.01

To screen novel drivers from oncogenic lncRNAs, we first performed analysis to identify overexpressed lncRNAs in LUAD, and a total of 596 lncRNAs were revealed based on TCGA LUAD datasets (Table S4). According to the previous report,^7^ we considered the association between gene expression and focal SCNA, amplification frequency, and clinical prognosis to discover novel LUAD noncoding drivers (Figure 1A; Table S4). Correlation analysis indicated a total of 175 lncRNAs that got positive correlation between gene expression and focal SCNA (Figure 1B). Among these candidate noncoding drivers, four of them were in high ranking according to amplification frequency, clinical relevance, and the correlation between expression and focal SCNA (Figure 1C). We subsequently used zebrafish tumor models to quickly validate the biological functions of these top ranked lncRNAs in vivo (Figure 1D). Approximately 400 DiI‐red–labeled A549 cells of each group were injected into the perivitelline space of each zebrafish embryo, and tumor cell proliferation and metastasis in the zebrafish body were monitored at 96 h postinjection (Figure 1E). Injection of A549 cells resulted in proliferation and dissemination of tumor cells from the primary sites, and the silence of candidate lncRNAs impaired these malignant phenotypes of A549 cells (Figure 1F). Among these lncRNAs, FAM83H‐AS1 was shown to exhibit the most significant effect in promoting proliferation and metastasis (Figure 1F). Intriguingly, previous studies indicated that FAM83H‐AS1 could act as the prognostic marker in types of cancer.^17^, ^18^, ^19^

### 3.2 Genomic amplification of FAM83H‐AS1 leading to poor prognosis of LUAD

**FIGURE 2:**
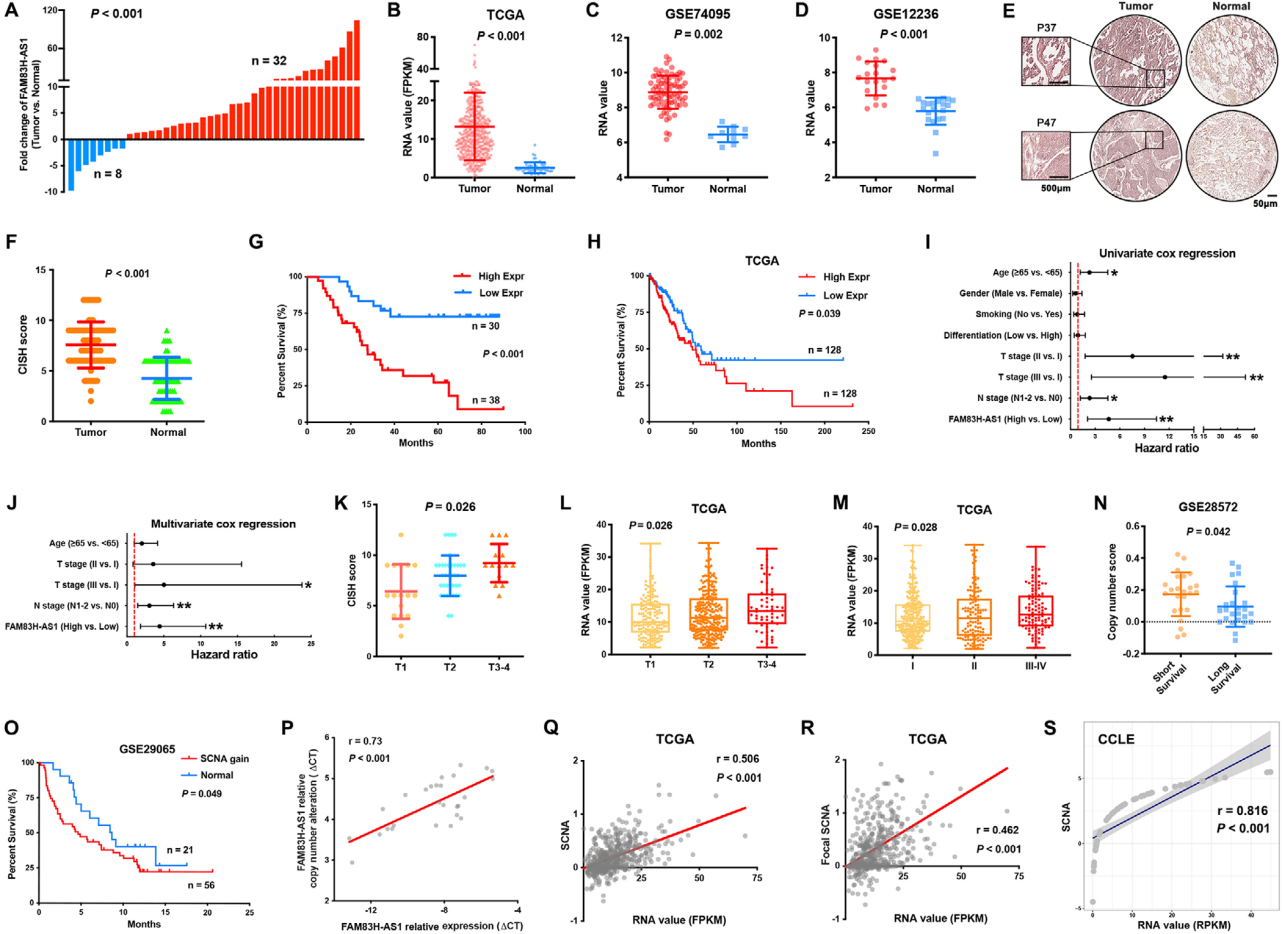
Clinical relevance of FAM83H‐AS1 in LUAD. (A) qRT‐PCR indicated that FAM83H‐AS1 was upregulated in 32 of 40 paired primary LUAD tissues in comparison to adjacent nontumor tissues. (B–D) Other independent datasets suggested the overexpression of FAM83H‐AS1 in LUAD tissues compared with adjacent nontumor tissues. FPKM, fragments per kilobase of exon model per million reads mapped. (E and F) The CISH results of the TMA of JSCH cohort. (G) Higher expression (>7, median) of FAM83H‐AS1 is associated with poor prognosis in JSCH cohort. (H) Higher expression (>17.5, quartile) of FAM83H‐AS1 got better prognosis than low expression (<7.25, quartile) in TCGA cohort. (I and J) Cox proportional hazards model indicated that the expression level of FAM83H‐AS1 was an independent prognostic factor. The horizontal line indicates the HR; and the vertical line includes data within 95% confidence interval. *p < 0.05 and **p < 0.01. (K) The expression level of FAM83H‐AS1 was positively correlated with tumor size in JSCH cohort. (L and M) The expression level of FAM83H‐AS1 was positively correlated with tumor size and TNM stages in TCGA cohort. (N and O) The high amplification level of FAM83H‐AS1 was found to be associated with poor prognosis in GSE28572 and GSE29065 datasets. (P) qRT‐PCR results demonstrated a high correlation between gene expression and amplification levels of FAM83H‐AS1 in LUAD tissues. (Q–S) Correlation analysis between the normalized expression and SCNAs of FAM83H‐AS1 in TCGA and CCLE datasets, respectively

We gained insights into the oncogenic functions of FAM83H‐AS1 in our LUAD cohort and other datasets. The expression of FAM83H‐AS1 was analyzed in 40 pairs of primary LUAD and adjacent nontumor tissues from the JSCH. FAM83H‐AS1 was highly upregulated in LUAD, with an average fold change of 13.30 (*p* < 0.01) (Figure 2A). TCGA and other datasets, including GSE74095 and GSE12236, all indicated that FAM83H‐AS1 was significantly overexpressed in LUAD tissues (Figure 2B–D).

The expression level of FAM83H‐AS1 was then detected in JSCH cohort by CISH using a TMA of 68 pairs of LUAD and adjacent nontumor tissues. Overexpression of FAM83H‐AS1 in LUAD was validated by CISH scores from the TMA (Figures 2E and 2F). Kaplan–Meier survival analysis showed that patients with a higher CISH score for FAM83H‐AS1 had a shorter OS (Figure 2G), and the result was validated by TCGA (Figure 2H). The Cox proportional hazards model indicated that FAM83H‐AS1 was an independent prognostic factor for LUAD (Figures 2I and 2J). Additionally, the expression level of FAM83H‐AS1 was positively correlated with tumor size and TNM stage in both JSCH and TCGA cohorts (Figures 2K and 2M). The copy number of FAM83H‐AS1 was shown to be related to poor prognosis in both the GSE28572 and GSE29065 datasets (Figures 2N and 2O). PCR results and TCGA data indicated the positive correlation between the expression and amplification levels of FAM83H‐AS1 (Figure 2P–R), and Cancer Cell Line Encyclopedia (CCLE) data validated these findings (Figure 2S).

### 3.3 The high expression level of FAM83H‐AS1 induced malignant behavior in LUAD cells

**FIGURE 3:**
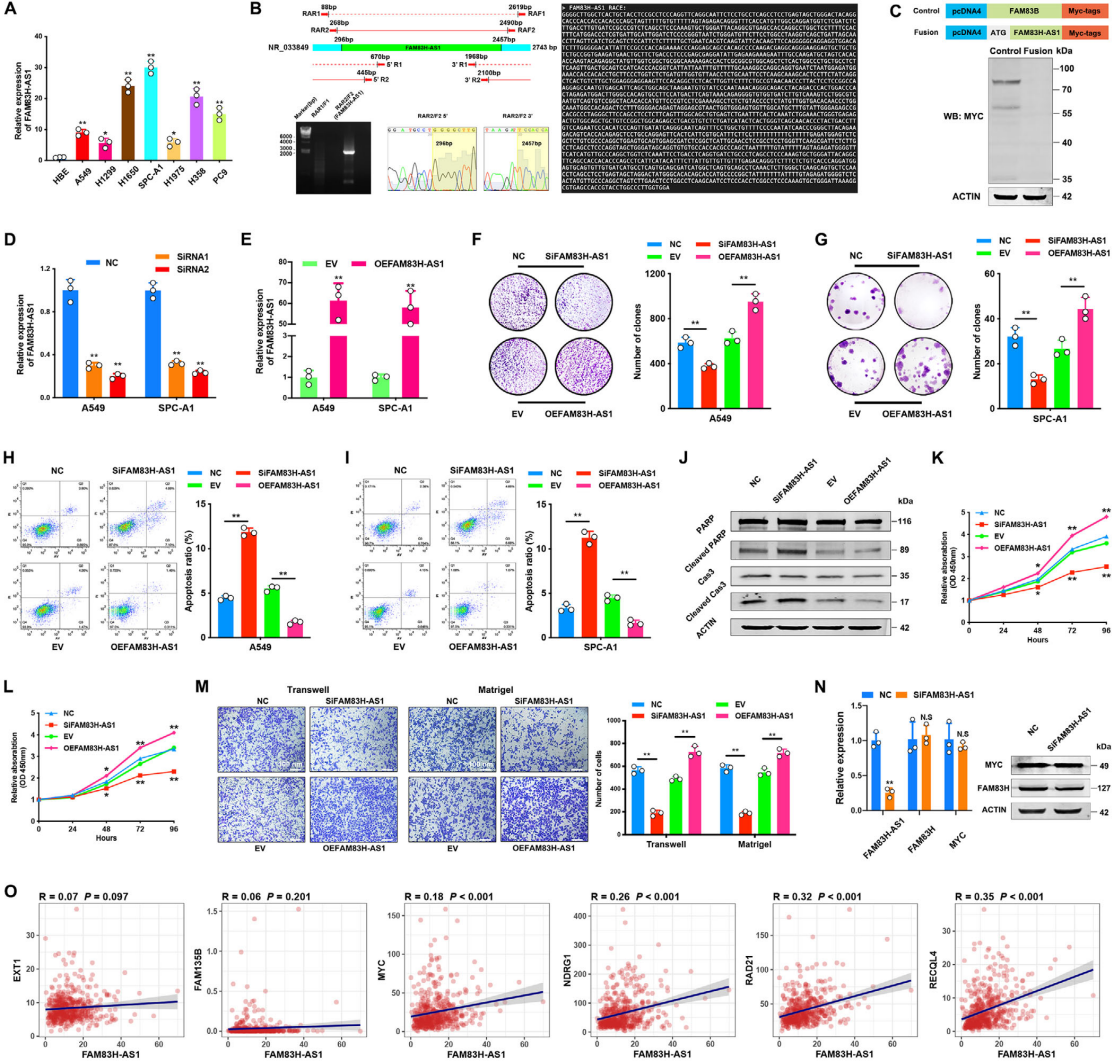
Features of FAM83H‐AS1 and gain and loss of function assays in vitro. (A) The expression of FAM83H‐AS1 was detected in seven LUAD and normal HBE cell lines. (B) The nested PCR product of RACE indicates an additional sequence of 295 nt in the 5′‐end and an additional 286 nt in the 3′‐end of reference NR_033849. Agarose gel and sanger sequencing assays demonstrated the full‐length of FAM83H‐AS1. (C) Coding potentiality assay indicated no translation of FAM83H‐AS1. Full‐length FAM83H‐AS1 was cloned into pcDNA4/myc‐HisB with an N‐terminal ATG codon and FAM83B served as a positive control. An anti‐MYC antibody was used to probe transcribed proteins. (D) SiRNA treatment reduced the expression level of FAM83H‐AS1 in both A549 and SPC‐A1 cells. (E) The efficiency of plasmid‐mediated overexpression of FAM83H‐AS1 in A549 and SPC‐A1 cells. (F and G) Silencing and overexpression of FAM83H‐AS1 suppressed and induced the clonogenicity of LUAD cells, respectively. (H–J) Apoptosis assay by flow cytometry and analysis of PARP and Caspase‐3 cleavage (A549). Knockdown of FAM83H‐AS1 greatly promoted apoptosis, whereas upregulation of FAM83H‐AS1 impaired the apoptotic ability of A549 and SPC‐A1 cells. Cas3, Caspase‐3. (K and L) CCK‐8 assay detected the proliferation of LUAD cells. Increase and decrease of FAM83H‐AS1 inhibited and facilitated the proliferation of A549 and SPC‐A1 cells, respectively. (M) Transwell and Matrigel assays showed that FAM83H‐AS1 regulated the migration and invasion abilities of LUAD cells, respectively. (N) qRT‐PCR and western blot demonstrated that FAM83H‐AS1 did not regulate driver gene MYC and head‐to‐head FAM83H. *p < 0.05 and **p < 0.01. (O) Correlation analysis between FAM83H‐AS1 and known driver genes (Cancer Gene Census) in 8q24 region based on TCGA data, and Pearson's correlation test was performed

The expression level of FAM83H‐AS1 was first detected in several LUAD cell lines and was found to be remarkably higher than it was in the normal bronchial epithelial cell line HBE (Figure 3A). FAM83H‐AS1 is located on chromosome 8q24 in human genome; a transcript length of 2162 nt was determined by 5′‐ and 3′‐RACE assays, which is slightly shorter than the reference transcript length of 2743 nt (NR_033849) (Figure 3B). Further, no translation of FAM83H‐AS1 was found according to coding potentiality assay (Figure 3C).

To investigate the biological function of FAM83H‐AS1, siRNA‐mediated knockdown and plasmid‐mediated overexpression of FAM83H‐AS1 were performed in LUAD cell lines, A549 and SPC‐A1, to explore its pathophysiological significance (Figures 3D and 3E). Colony formation assays showed that the silencing and overexpression of FAM83H‐AS1 suppressed and induced the clonogenicity of LUAD cells, respectively (Figures 3F and 3G). Using a flow cytometry assay, we determined that knockdown of FAM83H‐AS1 greatly promoted apoptosis, whereas ectopic expression of FAM83H‐AS1 impaired the apoptotic ability of A549 and SPC‐A1 cells (Figures 3H and 3I). Consistently, oncogenic functions of FAM83H‐AS1 were indicated by PARP and Caspase‐3 cleavage in apoptosis assays (Figure 3J). In addition, CCK‐8 assay results indicated that the knockdown and upregulation of FAM83H‐AS1 could obviously suppress and induce the proliferation of LUAD cells, respectively (Figures 3K and 3L). Transwell and Matrigel assays showed that FAM83H‐AS1 markedly affected the migration and invasion abilities of A549 and SPC‐A1 cells (Figure 3M).

### 3.4 FAM83H‐AS1 regulated LUAD cell apoptosis by binding HNRNPK

Although the head‐to‐head coding gene FAM83H has been known to be involved in the progression of human cancers,^20^, ^21^ we did not find any significant changes in FAM83H mRNA or protein in FAM83H‐AS1–reduced LUAD cells (Figure 3N, left panel). Additionally, the oncogene MYC, which is located in 8q24 and is known to be a driver in human cancers,^3^, ^22^ revealed no significant changes after the silencing of FAM83H‐AS1 (Figure 3N, right panel). Further analysis suggested weak correlation between FAM83H‐AS1 and all known driver genes in 8q24 region (Figure 3O), which indicated that FAM83H‐AS1 may impact downstream genes in trans.

**FIGURE 4:**
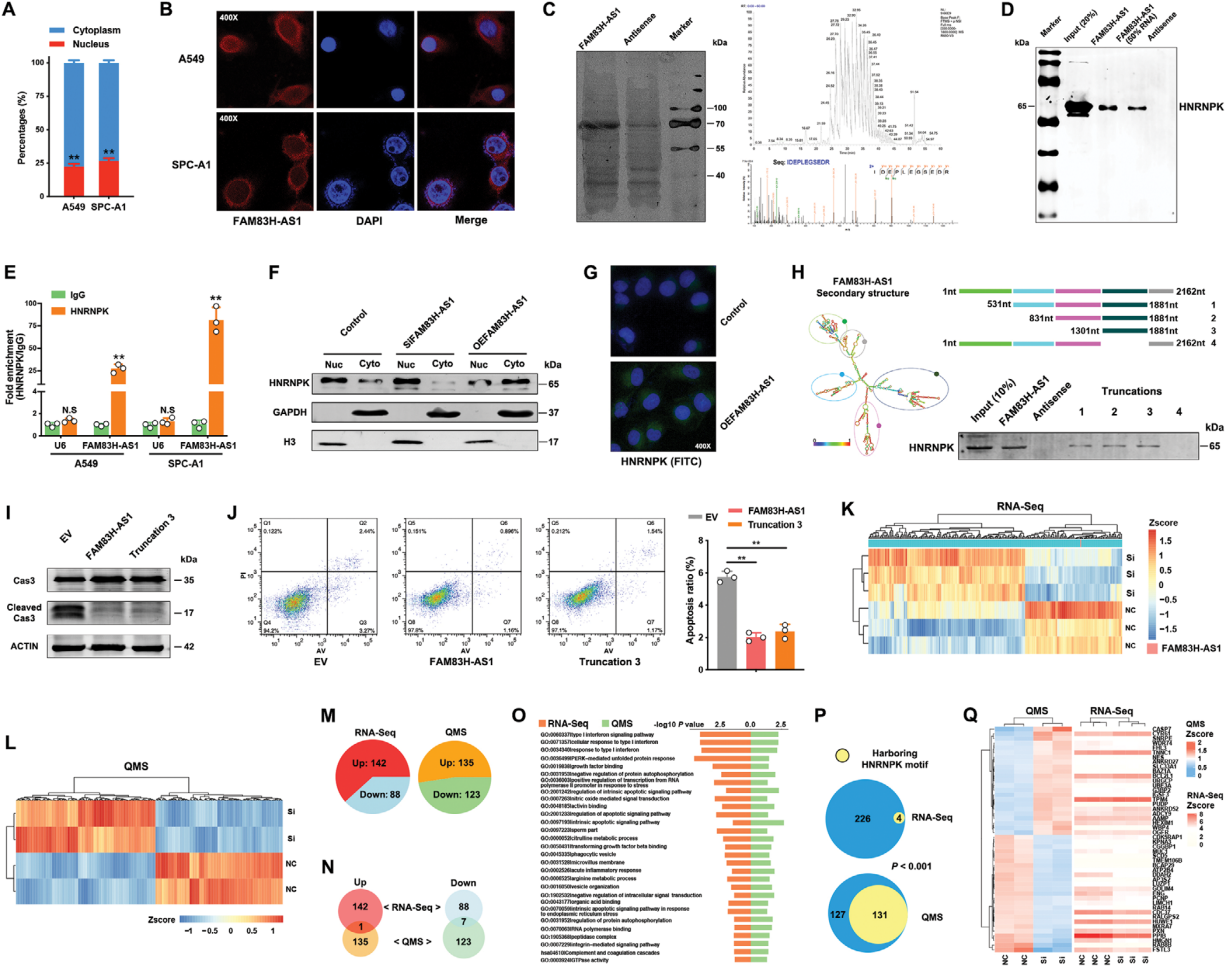
FAM83H‐AS1 binds HNRNPK to regulate LUAD cell apoptosis. (A and B) The nuclear mass separation and FISH assays suggested that FAM83H‐AS1 was mainly distributed in the cytoplasm. (C) RNA pull‐down and silver stain assays of biotinylated FAM83H‐AS1–associated proteins. The FAM83H‐AS1‐specific band was excised and analyzed by mass spectrometry, which identified HNRNPK. (D) Western blot of proteins from pulldown assays. (E) RIP evaluation of the interaction between HNRNPK and FAM83H‐AS1 using an anti‐HNRNPK antibody. IgG served as a negative control. U6, U6 small nuclear RNA (snRNA). *p < 0.05 and **p < 0.01; N. S., nonsignificant. (F) Western blot of HNRNPK expression in the cytoplasm and nucleus of the indicated A549 cells. Scrambled sequences and empty vectors were combined as controls. Empty vector and scrambled sequences were added to the silencing and overexpression groups, respectively. (G) Immunofluorescence assays indicated an increase in cytoplasmic HNRNPK after increasing the expression of FAM83H‐AS1 in A549 cells. (H) The secondary structure of FAM83H‐AS1 is shown as predicted by the centroid method (http://rna.tbi.univie.ac.at). The red color indicates strong confidence for the prediction of each base. RNA pull‐down detection of the interaction between HNRNPK and FAM83H‐AS1 truncations according to the predicted secondary structure. (I and J) Apoptosis and flow cytometry assays of cells with the full‐length and truncated FAM83H‐AS1, as assessed by Caspase‐3 cleavage. Cas3, Caspase‐3. (K–M) Differentially expressed genes identified by RNA‐Seq (FDR < 0.01 and fold change > 2) and QMS (*p* < 0.05 and fold change > 1.2) after silencing FAM83H‐AS1, respectively. (N) Common upregulated and downregulated genes in RNA‐Seq and QMS results. (O) Enrichment analysis based on Gene Ontology (GO) and Kyoto Encyclopedia of Genes and Genomes (KEGG) databases were performed on RNA‐Seq and QMS results, respectively, which revealed a total of 29 common pathways or biological processes (*p* < 0.05). (P) Differentially expressed genes harboring HNRNPK‐specific motifs in their 5′‐UTRs were identified in RNA‐Seq and QMS results. The *p*‐value was determined by Fisher's exact test. (Q) The top differentially expressed genes identified by QMS and corresponding mRNA changes in RNA‐Seq

To explore the molecular mechanisms of FAM83H‐AS1 in promoting LUAD tumorigenesis, we first performed nuclear mass separation and fluorescence in situ hybridization (FISH) assays. The results showed that FAM83H‐AS1 was mainly distributed in cell cytoplasm (Figures 4A and 4B), which indicated that FAM83H‐AS1 might exert biological function at the posttranscriptional level. A subsequent RNA pull‐down experiment was performed to identify potential proteins binding by FAM83H‐AS1 (Figure 4C, left panel). Mass spectrometry analysis on differentially displayed bands revealed that HNRNPK was associated with FAM83H‐AS1 (Figure 4C, right panel), which was then confirmed by western blot of the proteins isolated from the RNA pull‐down assays (Figure 4D). Additionally, a RIP assay was performed with an HNRNPK antibody to ensure that FAM83H‐AS1 formed a complex with HNRNPK (Figure 4E).

HNRNPK is an RNA‐binding protein that is localized both in the cytoplasm and nucleus,^23^ and it has been shown to regulate the translation of oncogenes in cancer cells.^24^, ^25^ Western blot assays showed that FAM83H‐AS1 did not affect the overall expression of HNRNPK within the A549 cells (Figure 4F); however, FAM83H‐AS1 overexpression increased HNRNPK expression within the cytoplasm, whereas FAM83H‐AS1 knockdown had an opposite effect (Figure 4F), which was buttressed using immunofluorescence assays (Figure 4G). Using biotinylated truncations of FAM83H‐AS1, we found that the stem‐loop structure from 1301 to 1881 nt (third truncation) was sufficient for enabling interaction between FAM83H‐AS1 and HNRNPK (Figure 4H). In addition, western blot demonstrated that the third truncation could suppress apoptosis at a rate similar to that of the full length FAM83H‐AS1 (Figures 4I and 4J).

### 3.5 FAM83H‐AS1 promoted the translation of RAB8B and RAB14

To identify the potential downstream targets of FAM83H‐AS1, we conducted RNA‐Seq and QMS assays after silencing FAM83H‐AS1 in A549 cells. A total of 230 differentially expressed mRNAs (FDR < 0.01 and fold change > 2) were detected (Figures 4K and 4M; Table S6), and the QMS assay demonstrated 258 differentially expressed proteins (*p*‐value < 0.05 and fold change > 1.2; Figures 4L and 4M; Table S7). Although we found that only a few differential genes overlapped at the mRNA and protein levels (Figure 4N), similar biological alterations after the silence of FAM83H‐AS1 were revealed by enrichment analysis between RNA‐Seq and QMS data (Figure 4O), including cell growth, interferon signaling pathways, and cell apoptosis. Considering that the FAM83H‐AS1 and HNRNPK complex is mainly distributed in the cytoplasm, we speculated that FAM83H‐AS1 may affect the translation of downstream genes in LUAD cells.

**FIGURE 5:**
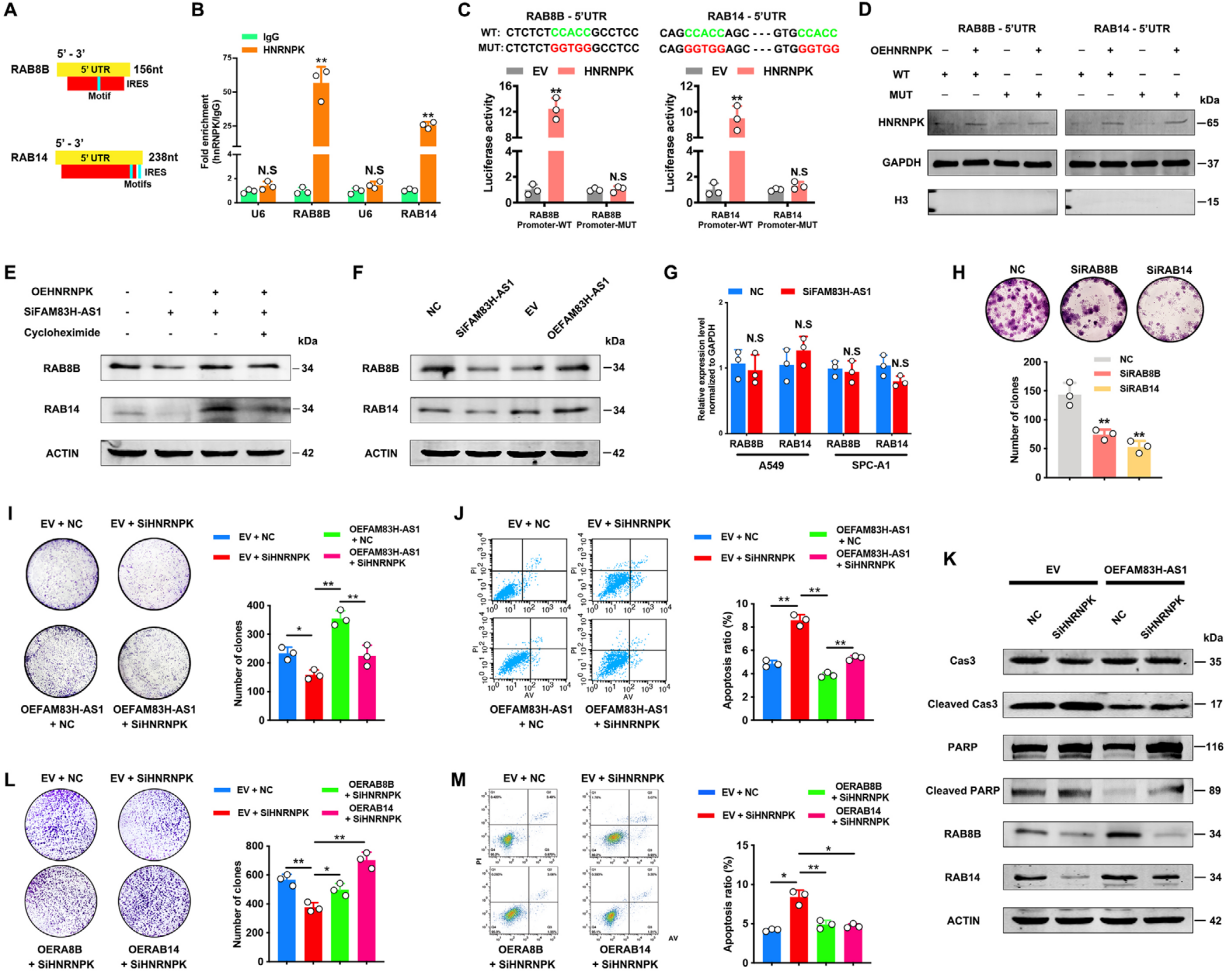
The FAM83H‐AS1–HNRNPK complex co‐regulates the expression of RAB8B and RAB14. (A) Predicted IRES sites (http://iresite.org) and identified HNRNPK motifs in the 5′‐UTRs of RAB8B and RAB14. (B) RIP evaluation of the interaction between HNRNPK and mRNAs of RAB8B and RAB14 using an anti‐HNRNPK antibody as described above. (C and D) Dual luciferase reporter assays showed that HNRNPK directly binds 5′‐UTRs of RAB8B and RAB14 and activates luciferase activity. Overexpressed cytoplastic HNRNPK in dual luciferase reporter assays of 293T cells. (E) Effect of the translation inhibitor cycloheximide on the HNRNPK overexpression‐induced increase in the protein levels of RAB8B and RAB14 in FAM83H‐AS1 knockdown A549 cells. (F) The silencing of FAM83H‐AS1 decreased the expression of RAB8B and RAB14, but overexpressing FAM83H‐AS1 increased their expression. (G) qRT‐PCR results revealed that RAB8B and RAB14 mRNAs were not regulated by FAM83H‐AS1. (H) The colony formation assay results suggested oncogenic functions for RAB8B and RAB14 in A549 cells. (I) Colony formation assays suggested that HNRNPK knockdown partially abolished the effects of FAM83H‐AS1. (J and K) Silencing HNRNPK reversed the effects of FAM83H‐AS1 on apoptosis, as revealed by flow cytometry and the cleavage of PARP and Caspase‐3, and the effect of HNRNPK knockdown on FAM83H‐AS1 overexpression‐induced protein expression of RAB8B and RAB14. Cas3, Caspase‐3. (L and M) Colony formation and flow cytometry assays indicated that the overexpression of RAB8B or RAB14 recovered the effects of HNRNPK, respectively. *p < 0.05 and **p < 0.01; N. S., nonsignificant

Since HNRNPK has been shown to have a preference for AU/CU‐rich sequences in 5′‐untranslated regions (UTRs) and to have a specified motif of N_2–5_C(C/U)ACC(C/A)N_11–17_.^26^ The differentially expressed genes harboring the HNRNPK motif in their 5′‐UTRs were identified in RNA‐Seq and QMS data (Figure 4P). Among the top ranked genes in the QMS results, no significant changes in expression level were revealed by RNA‐Seq (Figure 4Q). Two known oncogenes of the RAS family proteins, RAB8B and RAB14, were found to be significantly downregulated at the protein level after FAM83H‐AS1 silence. Considering the relative low RNA levels of RAB8B and RAB14 and the effect of HNRNPK in stimulating the activity of mRNA IRESs to regulate translation,^27^, ^28^ we analyzed the 5′‐UTRs of RAB8B and RAB14, which indicated potential IRES segments harboring HNRNPK motifs (Figure 5A). The RIP assays performed with HNRNPK antibody indicated that HNRNPK could bind RAB8B and RAB14 mRNAs (Figure 5B). Furthermore, we cloned the wild‐type and mutated 5′‐UTRs of the RAB8B and RAB14 mRNAs and performed dual luciferase reporter assays with them. Compared with the control group, the overexpression of HNRNPK efficiently promoted luciferase activity of wild‐type groups but not mutated groups (Figures 5C and 5D). These results suggested that the 5′‐UTRs of both RAB8B and RAB14 could be bound by HNRNPK. We also observed that the translation inhibitor cycloheximide inhibited the HNRNPK overexpression‐induced increase in the protein levels of RAB8B and RAB14 in FAM83H‐AS1 knockdown LUAD cells (Figure 5E).

The silencing of FAM83H‐AS1 decreased the expression of RAB8B and RAB14, whereas overexpressing FAM83H‐AS1 increased the expression of these genes only at the protein level (Figures 5F and 5G). The high expression level of RAB14 has been reported to inhibit apoptosis in NSCLC,^29^ and we found that the silencing of RAB8B or RAB14 suppressed the clonogenicity of A549 cells (Figure 5H). To determine whether FAM83H‐AS1 inhibits LUAD cell apoptosis via the FAM83H‐AS1–HNRNPK–RAB8B/RAB14 axis, colony formation, flow cytometry assay, and western blot of cleaved PARP and Caspase‐3 were performed; silence of HNRNPK partially rescued the apoptosis‐inhibiting effect induced by FAM83H‐AS1 (Figure 5I–K), and RAB8B or RAB14 could rescue the effect by silencing HNRNPK (Figures 5L and 5M). Additionally, the silence of HNRNPK partially reversed the effects of FAM83H‐AS1 on RAB8B and RAB14 (Figure 5K).

### 3.6 FAM83H‐AS1 could serve as a novel therapeutic target for LUAD

**FIGURE 6:**
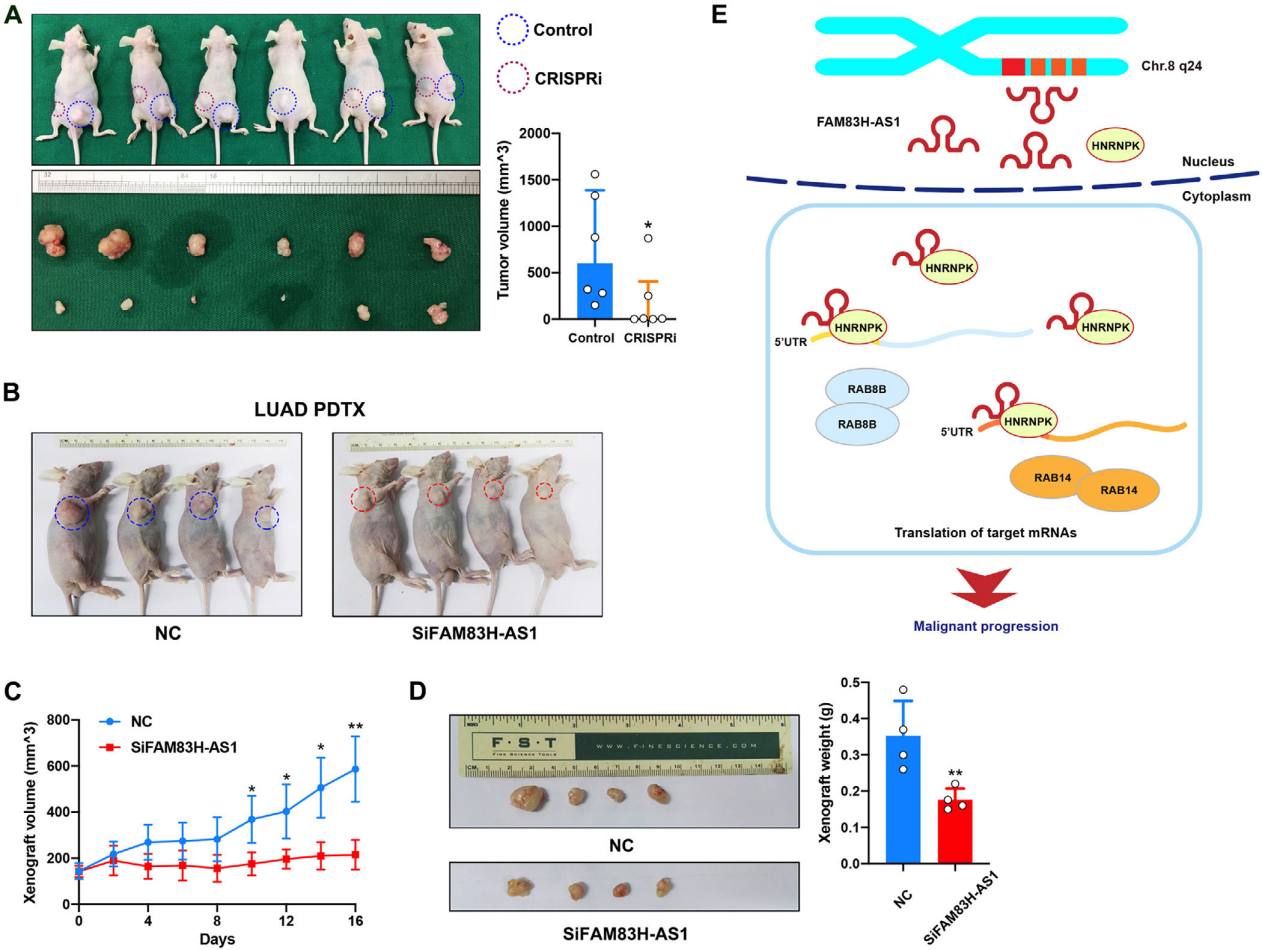
FAM83H‐AS1 promotes LUAD tumorigenesis in vivo and acts as a promising therapeutic target. (A) CRISPRi system‐constructed FAM83H‐AS1 knockdown A549 cells were incorporated in xenograft tumor models, which showed that tumors grown from FAM83H‐AS1 silenced cells were smaller than those grown from control cells. (B–D) PDTX model showing that intratumoral injection of siRNA targeting FAM83H‐AS1 inhibited LUAD‐derived tumor growth (four times and twice a week). *p < 0.05 and **p < 0.01. (D) Schematic diagram of how FAM83H‐AS1 promotes LUAD malignant progression. The chromosome 8q24 amplicon leads to overexpression of FAM83H‐AS1 in LUAD, and FAM83H‐AS1–HNRNPK complex binds mRNA 5′‐UTRs of RAB8B and RAB14 to promote translation

To validate the biological function of FAM83H‐AS1 in vivo, we constructed A549 cells with CRISPRi‐mediated FAM83H‐AS1 silencing. A total of six sgRNAs around the TSS of FAM83H‐AS1 were designed to suppress the transcription of FAM83H‐AS1 (Figure S1A), and the combination of three sgRNAs in the 3′‐end of TSS produced the highest knockdown efficiency (Figure S1B) without affecting the expression of head‐to‐head FAM83H (Figure S1C). Consequently, xenograft tumor models demonstrated that the tumors derived from CRISPRi‐mediated FAM83H‐AS1‐silenced A549 cells had a smaller tumor size than that of the control (Figure 6A).

We then developed a PDTX model from four LUAD patients and evaluated the therapeutic potential of targeting FAM83H‐AS1 by intratumoral injection of cholesterol‐conjugated siFAM83H‐AS1 and a control siRNA (Figure 6B). Immunohistochemistry revealed that the siFAM83H‐AS1 group had fewer RAB8B‐ and RAB14‐positive cells but more TdT‐mediated dUTP nick end labeling‐positive cells than the control group (Figure S1D). As a result, suppressing FAM83H‐AS1 inhibited PDTX growth in vivo (Figures 6C and 6D), suggesting that FAM83H‐AS1 could serve as a promising therapeutic target for LUAD.

## 4 DISCUSSION

In this study, we identified novel LUAD drivers from oncogenic lncRNAs using multi‐omics methods. The oncogenic functions of four candidate lncRNAs located at 8q24 region were tested in vivo using zebrafish models, which highlighted FAM83H‐AS1. In LUAD cells, FAM83H‐AS1 interacted with cytoplasmic HNRNPK to form an RNA–protein complex, which further regulated RAB8B and RAB14 by binding 5′‐UTRs of mRNAs. This interaction enhanced the translation of these oncogenes and upregulated their protein levels, which finally promoted the malignant progression of LUAD (Figure 6E).

To identify noncoding drivers in cancers, genomic variation‐associated data have been widely used in the discovery phase.^5^, ^6^, ^30^, ^31^ Integrative analysis of genomic and transcriptomic data provided a theoretical basis for identifying these candidate driver genes. Unlike coding genes, noncoding RNAs have been demonstrated to lack hotspot point mutations, but structural variants, including SCNAs, breakpoints, and fusion events, have been thought to be substantial contributors to noncoding drivers.^32^ High frequent SCNA gain or loss in cancer genomes has now been revealed by TCGA project, and joint analyses were performed on lncRNAs in several cancer types, including glioblastoma multiforme, ovarian cancer (OVCA), lung squamous cell carcinoma, and prostate cancer.^30^ Additionally, several lncRNAs, including RBPMS‐AS1, TDRKH‐AS1, LINC00578, RP11‐470 M17.2, and LINC00941, were revealed to be key prognostic markers of LUAD as a result of weighted gene co‐expression and SCNA analyses, but none of these lncRNAs have been validated in functional assays.^31^ In LUAD, chromosome 8q24 is a region with frequent SCNAs regardless of arm or focal levels,^33^ and it was also found to harbor most of candidate lncRNAs in our study. This so‐called “8q24 gene desert” was shown to be a hotspot region linking oncogenic lncRNAs and genomic variations,^34^, ^35^, ^36^ and this study added further insights into the oncogenic function of the 8q24 amplicon in LUAD.

Previous studies demonstrated that FAM83H‐AS1 has potent tumor‐promoting activity in colorectal carcinoma, breast cancer, bladder cancer, and NSCLC,^17^, ^18^, ^19^, ^37^ and the overexpression of FAM83H‐AS1 was also shown to be correlated with poor prognosis in OVCA and gastric cancer patients.^38^, ^39^ All these results indicated that FAM83H‐AS1 has conserved oncogenic function among different types of malignant tumors, even though the expression level varies greatly. Zhang et al. found that the proliferation, migration, and invasion of NSCLC cells were decreased after FAM83H‐AS1 downregulation,^37^ which is consistent with our findings. They also demonstrated the relationship between FAM83H‐AS1 and the MET/EGFR signaling pathway. EGFR pathway is the most common therapeutic target in NSCLC, and the status of EGFR pathway revealed the viability of cancer cells.^40^ Therefore, once the malignant phenotype of NSCLC cells was impaired after the downregulation of FAM83H‐AS1, the activation of EGFR pathway would be less intense accordingly. Although this finding revealed the potentiality of FAM83H‐AS1 to be a novel target in tyrosine kinase inhibitor‐targeted therapeutics, the specific mechanism behind this phenomenon is still needed to be further investigated.

Our findings indicated that FAM83H‐AS1 regulates downstream target genes that rely on HNRNPK, which is a multifunctional protein that plays important roles in cancer cells. Previous studies found that HNRNPK could regulate biological processes at both transcriptional and posttranscriptional levels. For example, HNRNPK was shown to interact with the RNA polymerase II transcription machinery to stimulate transcription^41^, ^42^ and be also involved in regulating the translation of MYC, P21, and ERK in cancer cells.^24^, ^25^, ^43^ Additionally, HNRNPK was found to be essential for the antiapoptosis mechanism in cancer cells, which is independent of P53 status.^44^, ^45^ Furthermore, HNRNPK protein has been revealed to play a regulatory role in the molecular mechanisms of lncRNAs.^46^ HNRNPK is required for lncRNA XIST‐mediated chromatin modifications^47^ and binds lncRNA CASC11 and LINC00460 to form RNA‐protein complexes in colorectal and lung cancers, respectively.^48^, ^49^ In the current study, we identified a complex of FAM83H‐AS1 and HNRNPK in LUAD cells. To control possible confounding bias, we used high‐throughput methods at both the RNA and protein levels to elucidate the underlying molecular mechanisms.

In conclusion, this study identified FAM83H‐AS1 as a potential noncoding driver of LUAD. We described the regulatory function of FAM83H‐AS1 on malignant phenotypes, especially for apoptosis and clonogenicity, and we revealed molecular mechanisms by which that FAM83H‐AS1 promoted the translation of antiapoptotic RAB8B and RAB14 by interacting with HNRNPK. All these findings offer a novel therapeutic strategy for LUAD by targeting oncogenic lncRNA FAM83H‐AS1.

## ETHICS APPROVAL AND CONSENT TO PARTICIPATE

This study was approved by the Medical Ethics Committee of Nanjing Medical University Affiliated Cancer Hospital. All animal studies were complied with the ARRIVE guidelines and conducted in accordance with the UK Animals (Scientific Procedures) Act, 1986 and associated guidelines, EU Directive 2010/63/EU for animal experiments, or the National Institutes of Health guide for the care and use of Laboratory animals (NIH Publications No. 8023, revised 1978). Experiments involving humans were in accordance with the ethical standards of committees (institutional and national) and with The Code of Ethics of the World Medical Association (Declaration of Helsinki). All patients completed written informed consent prior to study entry.

## CONFLICT OF INTEREST

The authors declare no conflict of interest.

## AUTHOR CONTRIBUTIONS

WSW and YR conceived and designed the study. HCC, LTY, MZF, QMT, WJ, ZXF, XWZ, XWJ, XYT, and HJW contributed to carry out the experiments. WSW and HCC contributed to data analysis. LTY and XL provided clinical samples and clinical information. WSW wrote the manuscript. YR and XL supervised the research. All authors read and approved the final manuscript.

## Supporting information

Supporting Information

Supporting Information

Supporting Information

## Data Availability

RNA‐Seq data have been submitted to GEO database and can be accessed with the ID: GSE159559. Most data relevant to the study are included in the article or uploaded as the Supporting Information. Others are available on request from the corresponding author.
