# Supplementary material for: FAM83H‐AS1 is a noncoding oncogenic driver and therapeutic target of lung adenocarcinoma
Source: Clin Transl Med. 2021 Feb 14;11(2):e316. doi: 10.1002/ctm2.316 (PMC7882096; doi:10.1002/ctm2.316)
Supplement: Supplementary file 1 — Supporting Information [file CTM2-11-e316-s003.docx]

**FAM83H-AS1 is a non-coding oncogenic driver and therapeutic target of lung adenocarcinoma**

**Siwei Wang, Chencheng Han, Tongyan Liu, Zhifei Ma *et al.***

**Supplementary Figure 1. CRISPRi and immunochemistry assays *in vivo***. A, For the use of CRISPRi system, sgRNAs were designed to target near the TSS of FAM83H-AS1. B, The combination of 3’-end sgRNAs had a higher knockdown efficiency than that of other experimental groups. C, Neither the 5’- nor 3’-end sgRNA combinations has an effect on the expression of the head-to-head FAM83H. D, Hematoxylin-eosin (HE) and TUNEL staining and immunochemistry staining of Ki67, RAB8B and RAB14 in PDTX models suggested that FAM83H-AS1 regulates the expression of RAB8B and RAB14 *in* *vivo*. *, *P* < 0.05, and **, *P* < 0.01. N.S, non-significant.

**Supplementary Table 1. Sequences of primers used for PCR in this study.**

**Supplementary Table 2. Primary antibodies or assay kits used in this study.**

**Supplementary Table 3. Sequences of siRNAs used in this study.**

**Supplementary Table 4. Analyses of uni- and multi-variate Cox regression in TMA results.**

**Supplementary Table 5. Genomic and prognostic features of identified 596 LUAD upregulated lncRNAs.**

**Supplementary Table 6. The significant differentially expressed genes identified by RNA-Seq after silencing FAM83H-AS1 in A549 cells.**

**Supplementary Table 7. QMS detected significant differentially expressed proteins in FAM83H-AS1 silenced A549 cells.**
