# Supplementary figures and images for: FAM83H‐AS1 is a noncoding oncogenic driver and therapeutic target of lung adenocarcinoma
Source: Clin Transl Med. 2021 Feb 14;11(2):e316. doi: 10.1002/ctm2.316 (PMC7882096; doi:10.1002/ctm2.316)

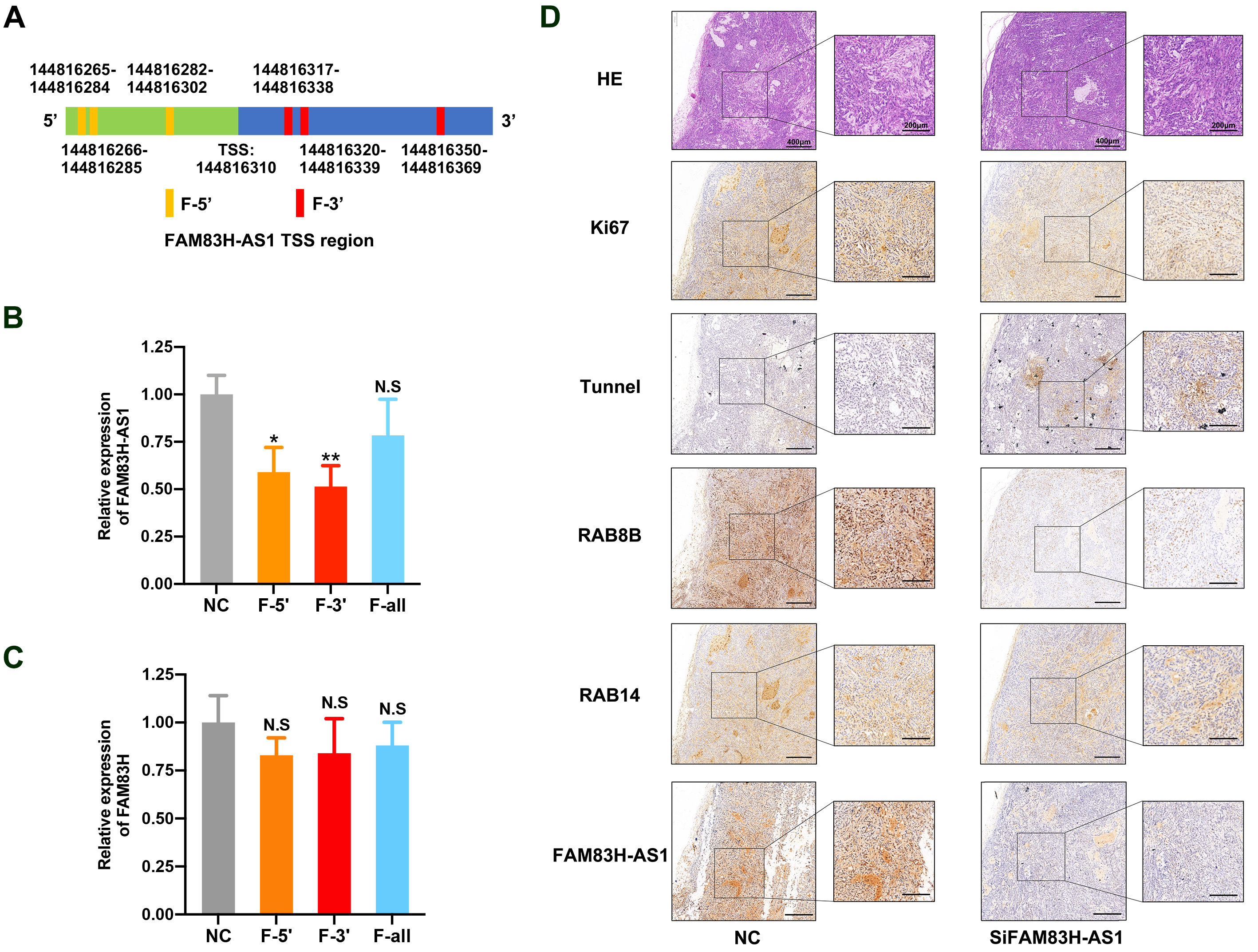

Supplement: Supplementary file 2 — Supporting Information [file CTM2-11-e316-s001.jpg]
